# Methodology for wave power estimation at remote sites with satellite altimeter validation applied to Indonesia

**DOI:** 10.1007/s40722-025-00455-0

**Published:** 2025-12-08

**Authors:** Jason McIlvenny, Benjamin J. Williamson

**Affiliations:** University of the Highlands and Islands, Inverness, UK

**Keywords:** Wave energy, Sumbawa, West Nusa Tenggara, Resource characterisation

## Abstract

This study investigated wave power at a remote site in Indonesia where in-situ data are scarce to inform feasibility and design parameters for a modular wave energy converter. The methodology was developed using models to assess initial wave energy, and to validate these models using remote-sensing data in the absence of in-situ observational data, intended to also be used more widely in data-poor locations to provide an assessment of wave energy site suitability. To assess the available wave energy, two models were employed: the Copernicus WAVERYS global wave model and a high-resolution model using open-source hydrodynamic modelling software. Affordable bathymetric data were used, and due to the lack of in-situ wave data for validation, the model’s precision was confirmed by comparing it with the existing satellite altimeter data. The high-resolution model indicated a redistribution of wave energy around the target area in the southern region of Sumbawa Island, revealing an average wave energy potential of 18 kW/m over a decade at the location. The analysis showed the area to be suitable for this demonstration project, and the methods used here could be employed at other remote sites in the Indonesian archipelago.

**Supplementary Information:**

The online version contains supplementary material available at 10.1007/s40722-025-00455-0.

## Introduction

Indonesia, with its vast archipelago, possesses abundant renewable energy resources. The Indonesian government has set ambitious targets to derive a substantial portion of its energy from these resources, increasing its renewable energy share to 25% by 2025 and 44% by 2030 (BPPT 2016; IRENA 2022). Amongst them, wave energy stands out as a clean and highly available option (Silva et al. 2015), with the island chain having over 4000 km of southern facing shorelines open to reliable long-period swell generated in the Indian Ocean. Addressing in-land deforestation for fuel and reducing reliance on fossil fuels are critical for mitigating emissions and climate concerns. By harnessing near-infinite wave energy resources along its extensive coastlines, Indonesia could potentially create a marine energy market, generating new jobs and saving million of tonnes of CO_2_ (Sugianto et al. 2017).

The geography of the archipelago makes access to traditional energy resources difficult in some remote, underdeveloped areas (Lasabuda 2013); in such places, small-scale wave energy converters (WEC) could play a key part in electrical supply, including providing energy security, offsetting diesel generation, and increasing access to electricity. Whereas Java has nearly universal electrification, Nusa Tenggara struggles to provide reliable electricity access to millions of households. This is exacerbated in remote rural areas of eastern Indonesia which also have difficulties attracting infrastructure investment. Simultaneously, the local use of diesel generators worsens air quality, increases greenhouse gas emissions, and increases vulnerability to fossil fuel price volatility (Asian Development Bank 2020). The combination of remote geographical circumstances and the high, fluctuating cost of diesel fuel create an ideal location for wave energy which is abundant in such coastal localities (Amiruddin et al. 2019).

Such remote village areas may benefit from small-scale, modular wave energy converters which are relatively simple and easy to maintain (Foteinis 2022). Recent studies show that a high wave energy potential of over 20 kW/m is available year-round for many locations (Ribal et al. 2020, Irhas and Suryaningsih, 2014; Rizal et al. 2020; Amiruddin et al. 2019). Such studies have used global reanalysis models such as WAVEWATCH III (Ribal et al. 2020) to derive their estimates.

This study focuses on such an area on the Island of Sumbawa, on the southwest shoreline in the West Nusa Tenggara province, where a high-resolution wave model was developed to estimate the suitability of the site for such a small-scale wave energy converter, providing a case study for the wider challenge of assessing renewable energy resource to support decarbonisation of remote locations in data-sparse areas.

There are numerous designs of WEC with various advantages and disadvantages and suitability to different locations and conditions, such as oscillating water column, overtopping, and pressure differential, for example (Thomas and Prakash 2020). The design considered here is a relatively simple point absorber due to minimal environmental impact and designed for maximum efficiency with ease of maintenance which is of great importance in remote locations. Here, the design is to place a buoy in the water column with a line anchored to the seabed and have the required infrastructure for electricity generation based on land (Song et al. 2017). Due to multi-directional energy harvesting capabilities and low-maintenance infrastructure, this device can operate effectively at significantly lower wave energy levels than conventional WECs, such as larger systems which typically require a 10-year mean wave power exceeding 20 kW/m (Bouhrim et al. 2024). In contrast, this system is designed to function efficiently in environments with a 10-year mean wave power as low as 5–10 kW/m (INGINE Ltd, Song et al. 2017). The development is aiming to provide a 125 kW wave power plant at the site.

For assessing wave energy availability in this region, two wave models were used: a global model for which data are readily available and a small-scale high-resolution model which was developed for a site of interest. In-situ wave data for calibration and validation of the models were not available within the site of interest or model extent, and high-resolution bathymetry is not available for many parts of Indonesia. These problems were overcome using a low-cost bathymetric survey of the site of interest and a combination of available bathymetry. Satellite altimeter data were used to check the validity of the models in the site of interest.

## Methods

Many suitable candidate sites were initially considered for the wave energy converter; however, the candidate site was selected based on many different factors which would lead to an effective installation, such as available infrastructure, energy requirements of local villages nearby, ease of access, and low environmental impacts.

**Fig. 1:**
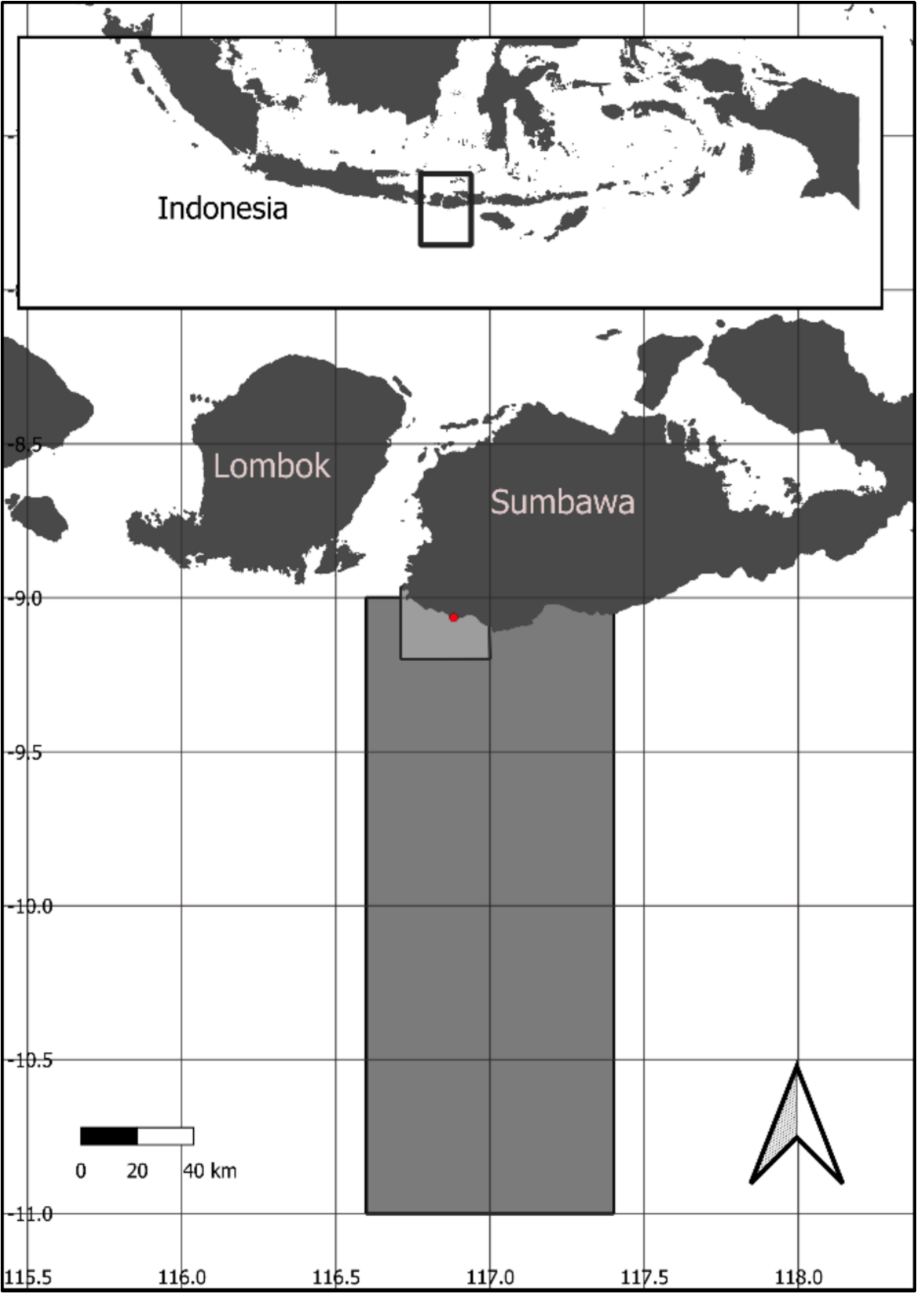
Map of area of interest of the southwest coast of Sumbawa. Large grey box indicates extent of data extracted from Copernicus WAVERYS model. Lighter smaller rectangle indicates extent of high-resolution TELEMAC model. Red dot indicates site of interest near Hasama Cliff

The site of interest for the demonstration project is in the southeast of West Nusa Tenggara, offshore from Hasama Cliff, Punak beach (− 9.0618° Lat., 116.8823° Lon., Fig. 1).

The models aimed to estimate seasonal, annual, and decadal wave power, informing the design of a small modular wave energy converter tailored to site conditions. A global model was used to estimate offshore wave energy, and to provide data to drive the boundary conditions of a high-resolution model which would resolve wave conditions to the nearshore.

### Global model data

This study used the global wave reanalysis dataset supplied by Copernicus Marine Service with the identification of GLOBAL_REANALYSIS_WAV_001_032. This product also bears the name of Global Ocean Waves Reanalysis (WAVERYS) within the Global High Resolution-Monitoring and Forecasting Centre (GLO-HR MFC). The model extends from 1993 to 2021.

The core of WAVERYS is based on the Météo-France Wave Model (MFWAM), a third-generation wave model that calculates the wave spectrum. Average wave quantities derived from this wave spectrum, such as the significant wave height (SWH) or the average wave period, are delivered on a regular 1/5° grid with a 3-h time step, with the wave spectrum discretised into 30 frequencies. WAVERYS considers oceanic currents from the GLORYS12 physical ocean reanalysis and assimilates significant wave height observed from historical altimetry missions and directional wave spectra from Sentinel 1 SAR from 2017 onwards.

Data were requested for the following period and spatial extent:

- 10-year dataset; 3 hourly intervals. Start: 2010-01-01 00:00:00 and End: 2020-12-31 00:00:00
- Wave energy maps use all model grid points in box (Top Left) − 9° N, 116.6° E, (bottom right) − 11° N, 117.4° E (see Fig. 1).

For this first project stage, a statistical analysis of the data from the global model was undertaken resulting in wave statistics tables and overall available wave energy maps offshore from the site of interest.

### High-resolution wave modelling

For the second project stage, the open-source TELEMAC-Mascaret suite of numerical solvers, developed by EDF R&D’s Laboratoire National d’Hydraulique et Environnement (LNHE) (Galland et al. 1991), was used to derive a high-resolution wave model of the site of interest with wave parameters at the model boundaries given by the WAVERYS global reanalysis dataset in stage one of the project. The TOMAWAC module for wave propagation into shallow water was used. By means of a finite-element type method, it solves a simplified equation for the spectro-angular density of wave action.

The physical processes modelled comprise (a) energy source/dissipation processes (wind-driven interactions with atmosphere, dissipation through wave breaking/white capping/wave-blocking due to strong opposing currents, and bottom friction-induced dissipation); (b) non-linear energy transfer conservative processes (resonant quadruplet interactions, triad interactions); and (c) wave propagation-related processes (wave propagation due to the wave group/current velocity, depth-current induced refraction, shoaling, and interactions with unsteady currents). The models compute the evolution of wave action density N by solving the action balance equation (Booij et al. 1999; Ris et al. 1999; Samaras et al. 2016)

1 $$
\frac{\partial N}{\partial t}+{\nabla }_{x,y }\left[\left({C}_{g}+U\right)N\right]+\frac{\partial }{\partial \sigma }\left({C}_{\sigma }N\right)+\frac{\partial }{\partial \varnothing }\left({C}_{\varnothing }N\right)=\frac{{S}_{tot}}{\sigma },
$$

where *N* = *E*/*σ*, E being the variance density and *σ* the relative angular frequency, ${C}_{g}$ is the intrinsic group velocity vector, *U* is the ambient current, ${C}_{\sigma }$, ${C}_{\varnothing }$ are the propagation velocities in spectral space (*σ*, *θ*), and ${S}_{tot}$ is the source/sink term that represents all physical processes which generate, dissipate, or redistribute energy. Broken down to its components, ${S}_{tot}$ can be written as

2 $${S}_{tot}={S}_{in}+{S}_{wc}+{S}_{n14}+{S}_{bf}+{S}_{br}+{S}_{n13},$$

where ${S}_{in}$ represents the energy transfer from wind to waves, ${S}_{wc}$ the dissipation of energy due to whitecapping, ${S}_{n14}$ the non-linear transfer of energy due to quadruplet (four-wave) interactions, ${S}_{bf}$ the dissipation due to bottom friction, ${S}_{br}$ the dissipation due to wave breaking, and ${S}_{n13}$ the non-linear transfer of energy due to triad (three-wave) interactions. Further detailed information on all the equations used within the TOMAWAC module are provided in the TOMAWAC user manual available to download: http://wiki.opentelemac.org/doku.php?id=user_manual_tomawac. At each point of the computational mesh, TOMAWAC calculates the following information: significant wave height; mean wave frequency; mean wave direction; peak wave frequency; wave-induced currents; radiation stresses.

The wave field is introduced to the model domain at the boundaries computed as a spectrum of multi-directional random waves. The shape of the spectrum and direction parameters are given by the following parameters: type of boundary directional spectrum = 6 (JONSWAP spectrum), frequency ratio = 1.12, minimal frequency = 0.05, number of directions = 36, and number of frequencies = 17. Whereby the frequency distribution within the model domain is given by $Mo*{r}^{n}$, where $Mo$ is the minimal frequency and *r* is the frequency ratio. The number of directions is divided equally by 360/number of directions in degrees. The number of frequencies and directions used was based on computational efficiency and accuracy for producing realistic results within the model grid; the settings used are of that used in other studies using the TOMAWAC module (Zhang et al. 2023; Basu et al. 2024; Park 2009; Machado et al. 2021; Kirinus et al. 2018).

The significant wave height, peak frequency, and peak direction were supplied at the boundary nodes as a user-defined comma-delimited text file with each attribute read to build the spectrum at specific time steps, with significant wave height (HM0) in metres, peak frequency (FP) in Hertz, and direction in radians. To enable the reading of this file by TOMAWAC, a user-defined alteration to one of the Fortran scripts was required. The script LIMWAC.f was altered, so that this file would be read at predefined time steps. The type of boundary spectrum was set to JONSWAP. Other keywords which were activated were:

- Bottom friction dissipation (in accordance with WAM cycle 4), default coefficient (0.038)
- Depth-induced breaking dissipation (Battjes and Janssen’s model (Battjes and Janssen 1978))
- Nonlinear transfers between frequencies (LTA model interaction in accordance with WAM cycle 4, model proposed by Eldeberky and Battjes (1996)
- No consideration of wind.

The model extent was such that it extended to the position of the nearest grid points of the global model, approximately 17 km offshore from Sumbawa Island (Fig. 1).

Two positions in the site of interest were selected as potential sites for the WEC buoys, Buoy 1: − 9.06384° N, 116.88119° E, Buoy 2: − 9.063925° N, 116.88139° E (EPSG 4326—WGS84).

Data were extracted from the high-resolution model for statistical analysis for these positions. The model was run for a 10-year period (2010–2020) and temporal data extracted from the model at 15-min intervals.

The model mesh for the TOMAWAC model was generated using the Blue Kenue tool for hydraulic modellers (https://nrc.canada.ca/en/research-development/products-services/software-applications/blue-kenuetm-software-tool-hydraulic-modellers) (Barton 2019). The model mesh element sizes were controlled using a density file of points distributed throughout the model with a size variable.

**Fig. 2:**
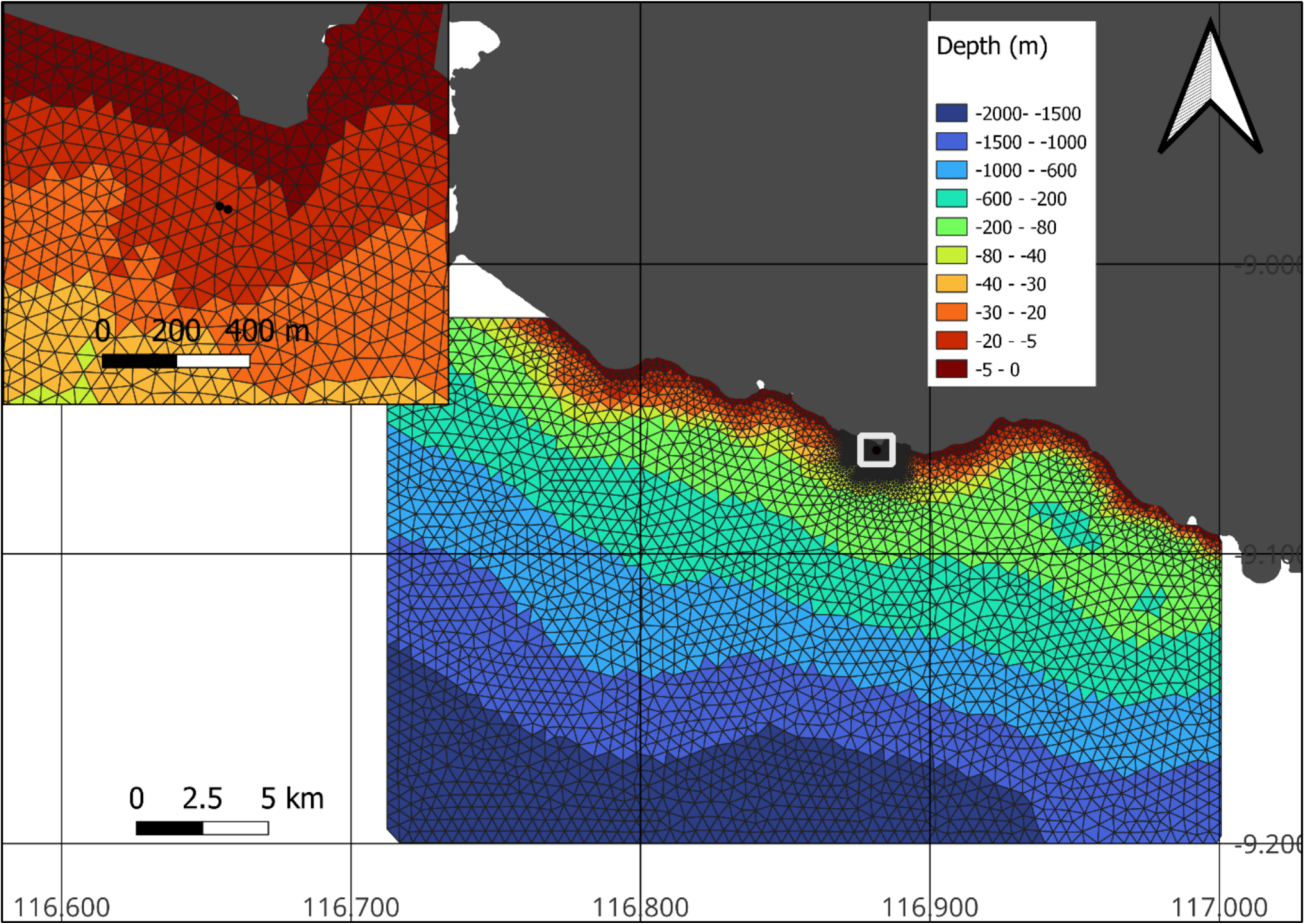
Model mesh and combined bathymetry

The size of the model area was determined from the nearest grid points from the global reanalysis dataset to the site of interest, approximately 17 km offshore, leading to a model area of approximately 31 km by 20 km. For computational optimization, the element size varied from 500 m at the boundaries (Fig. 2), to 100 m nearshore and 50 m in the site of interest (Fig. 2). The model mesh had a total of 4244 nodes with 7848 elements.

### Bathymetry data

Due to the lack of high-resolution bathymetric data in the model extent, the bathymetry for the model was derived from three sources.

1. Local bathymetry of the site of interest. The local bathymetry was derived from a bathymetric survey undertaken by project partners and supplied as point data (Fig. 2).
2. Bathymetry up to 1000 m deep outside the site of interest was derived from chart data from the Indonesian Navy Hydro-Oceanographic Centre (Pusat Hidro-Oseanografi TNI AL; Map Sheet 293).
3. GEBCO global bathymetry dataset > 1000 m (https://www.gebco.net/data_and_products/gridded_bathymetry_data/). Data supplied as point XY data.

A bathymetry survey was conducted using a single-beam echosounder (Telydyne Hydrotrack 11 single-frequency portable hydrographic echo sounder), mounted on a small RIB vessel. The survey measured water depth at regular horizontal and vertical intervals along the designated survey area. The collected data were then calibrated to locally obtained tidal elevation data, adjusting the depth measurements to low water spring as a reference. The three bathymetric data sources were merged to produce a final bathymetry set for the model input. The merged bathymetry was interpolated onto the model mesh via linear interpolation (Fig. 2). Potential errors in the wave model could occur from this methodology, in particular in the nearshore where some features could not be accounted for; however, by undertaking the dedicated mapping of the bathymetry within the nearshore area of the site of interest, these errors were minimised.

No calibration was undertaken for the high-resolution model; due to remoteness of the site, no in-situ observational wave data were available. However, both models were compared against AltiKa altimeter data; the AltiKa altimeter on board the SARAL satellite has a small footprint providing good quality measurements closer to land than previous space-based altimeters (Goddijn-Murphy et al. 2015), providing excellent accuracy even within 2 km of the coastline (Hithin et al. 2015; Bhowmick et al. 2025; Uti et al. 2018). The satellite returns to the same location with a temporal resolution of 35 days. Wave heights surrounding 1 km of the model grid points were compared and with a temporal shift of no more than 1.5 h. For the high-resolution model, all available AltiKa derived significant wave height values within the model extent were compared with the nearest model grid point with a temporal shift of ± 15 min. Four statistical comparisons were made between the datasets, root-mean-squared error (RMSE), coefficient of determination (*R*^2^), bias (observed-predicted), and the scatter index, defined as the observed mean normalised root-mean-squared error expressed as a percentage.

Wave energy was calculated from the WAVERYS reanalysis model and the TELEMAC–TOMAWAC model. Wave energy was calculated as W/m by

3 $$P=\frac{\rho {g}^{2}{T}_{p}{{H}_{s}}^{2}}{64\pi },$$

where *P* represents the wave power per unit crest length, measured in Watts per metre (W/m). The variable ρ denotes the density of seawater, 1025 kg per cubic metre (kg/m^3^), whilst *g* is the acceleration due to gravity, approximately 9.81 m per second squared (m/s^2^). The wave period is represented by *T*_*p*_, measured in seconds (s), and *H*_*s*_ stands for the significant wave height in metres (m).

## Results

**Fig. 3:**
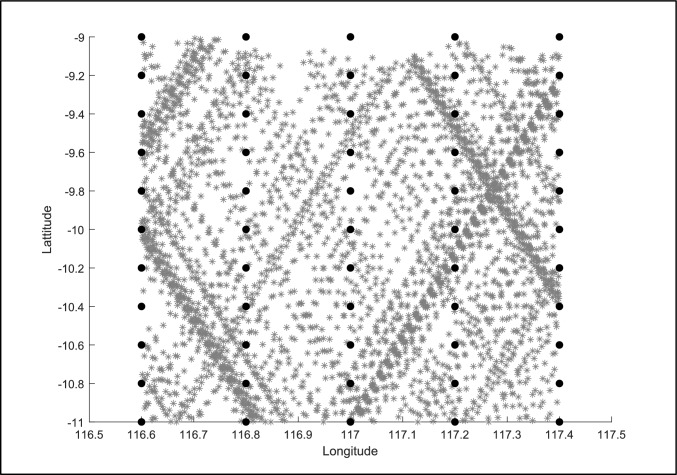
AltiKa satellite coverage 2014–2019 (grey star) and WAVERYS model points (black circles)

The WAVERYS model was compared to AltiKa satellite-derived wave height for a comparison of the model’s accuracy in the region of interest. Altimeter data within 1 km of each selected grid point were compared; all available altimeter data points and grid points are shown in Fig. 3.

**Fig. 4:**
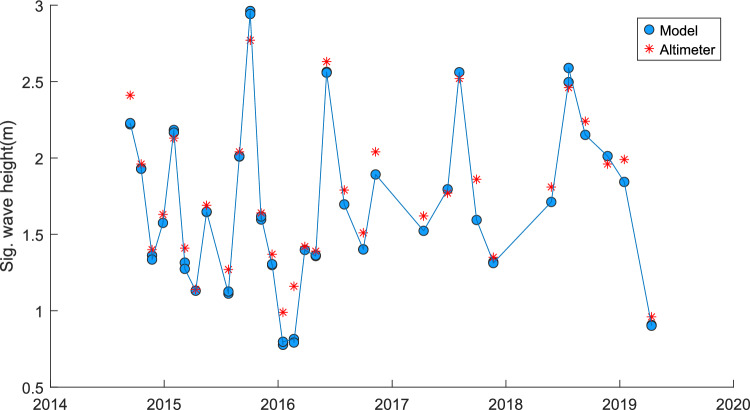
WAVERYS-derived significant wave height comparison against satellite altimeter data. − 9.2° Lat, 116.8° Lon. RMSE = 0.23, *R*^2^ = 0.79, bias = 0.13, scatter index = 13%

**Fig. 5:**
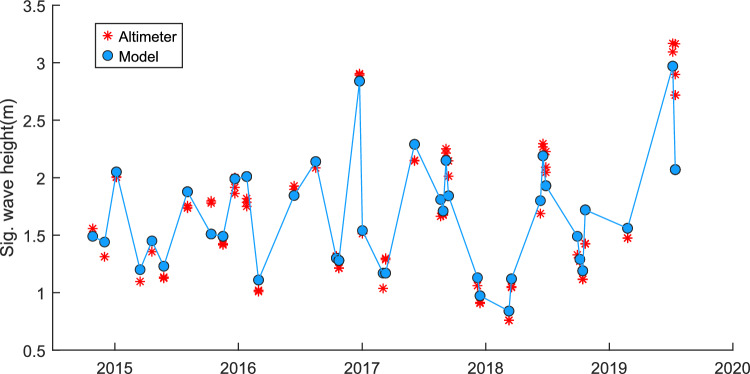
WAVERYS-derived significant wave height comparison against satellite altimeter data. − 11.0° Lat, 116.8° Lon. RMSE = 0.086, *R*^2^ = 0.97, Bias = 0.09, scatter index = 5%

**Fig. 6:**
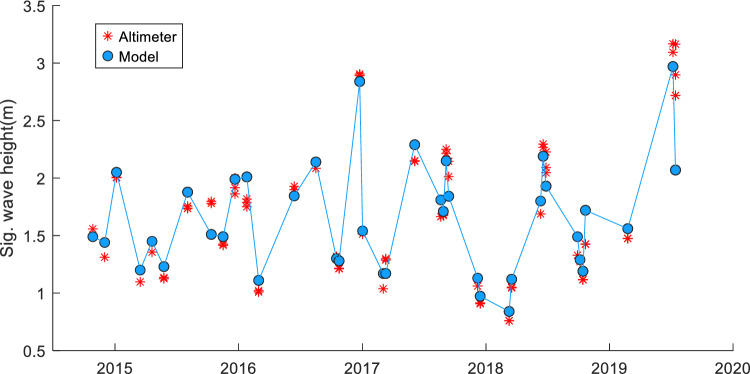
TOMAWAC-derived significant wave height comparison against satellite altimeter data. Points represent all satellite crossings over the high-resolution model area (− 9.2°, 116.7°, − 9.0°, 11.0°) available over the timeframe. RMSE = 0.23 m, *R*^2^ = 0.87, bias = 0.14, scatter index = 12%

Two selected WAVERYS grid points were selected for comparison, one point, which was used as model boundary data for the high-resolution model (− 9.2° Lat., 116.8° Lon., Fig. 4), and one at the edge of the selected global model extent for the site of interest (− 11.0°, 116.8°, Fig. 5). All AltiKa passes within the high-resolution TOMAWAC model domain, spatially and temporally, were compared to the model data (Fig. 6).

Comparison of AltiKa satellite observations, WAVERYS reanalysis data, and TOMAWAC model outputs demonstrates strong agreement, with high correlation and low scatter. This indicates that the WAVERYS boundary conditions are being accurately propagated and dynamically resolved within the high-resolution TOMAWAC model domain.

**Fig. 7:**
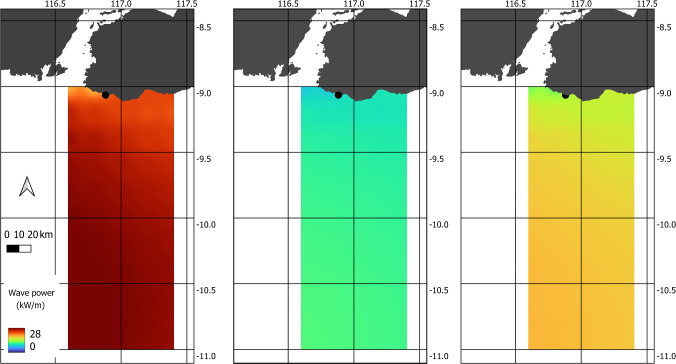
Seasonal wave power variation in WAVERYS linear interpolated model data, (left) mean July, (centre) mean December, and (right) 10-year mean. Black dot indicates site of interest position. Additional wave energy flux maps are presented in the Supplementary Information

**Fig. 8:**
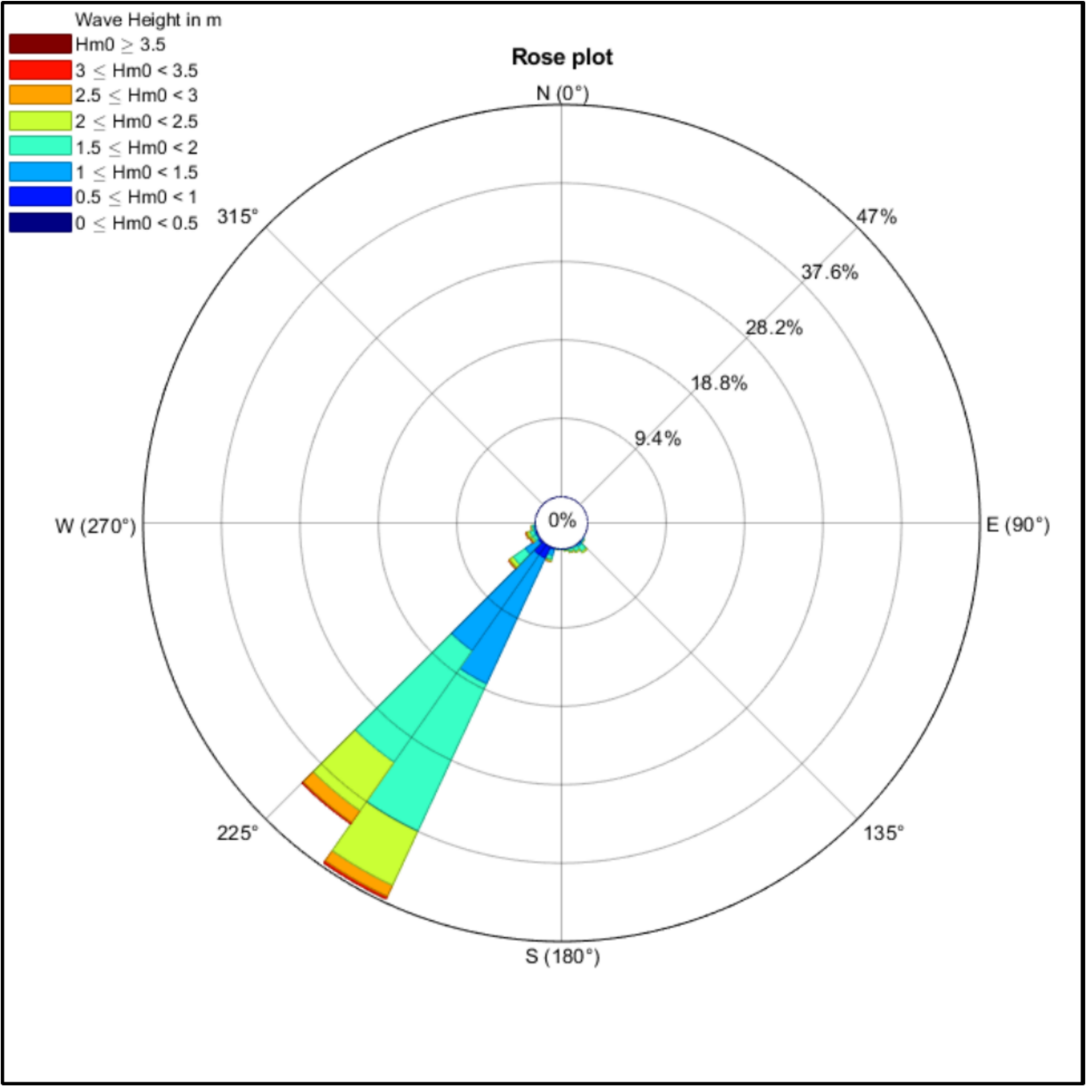
10-Year significant wave height direction plot for extracted WAVERYS coordinates

Average wave energy flux maps were produced from the statistical analysis of the global WAVERYS model. Maps were collated for both monthly averages, yearly averages and for the 10-year average from the whole dataset (Fig. 7). Other statistical tables and plots were also generated, including wave direction rose plots (Fig. 8).

The wave power plots from the WAVERYS analysis indicate that 10-year monthly mean values for this coastline seasonally vary from a maximum of 20 kW/m in July to a seasonal low of 8–10 kW/m during November/December (Fig. 7 and Supplementary Information). Mean wave direction is consistent from the southwest (coming from 225° to 205°, Fig. 8) and showed little seasonal variation.

**Fig. 9:**
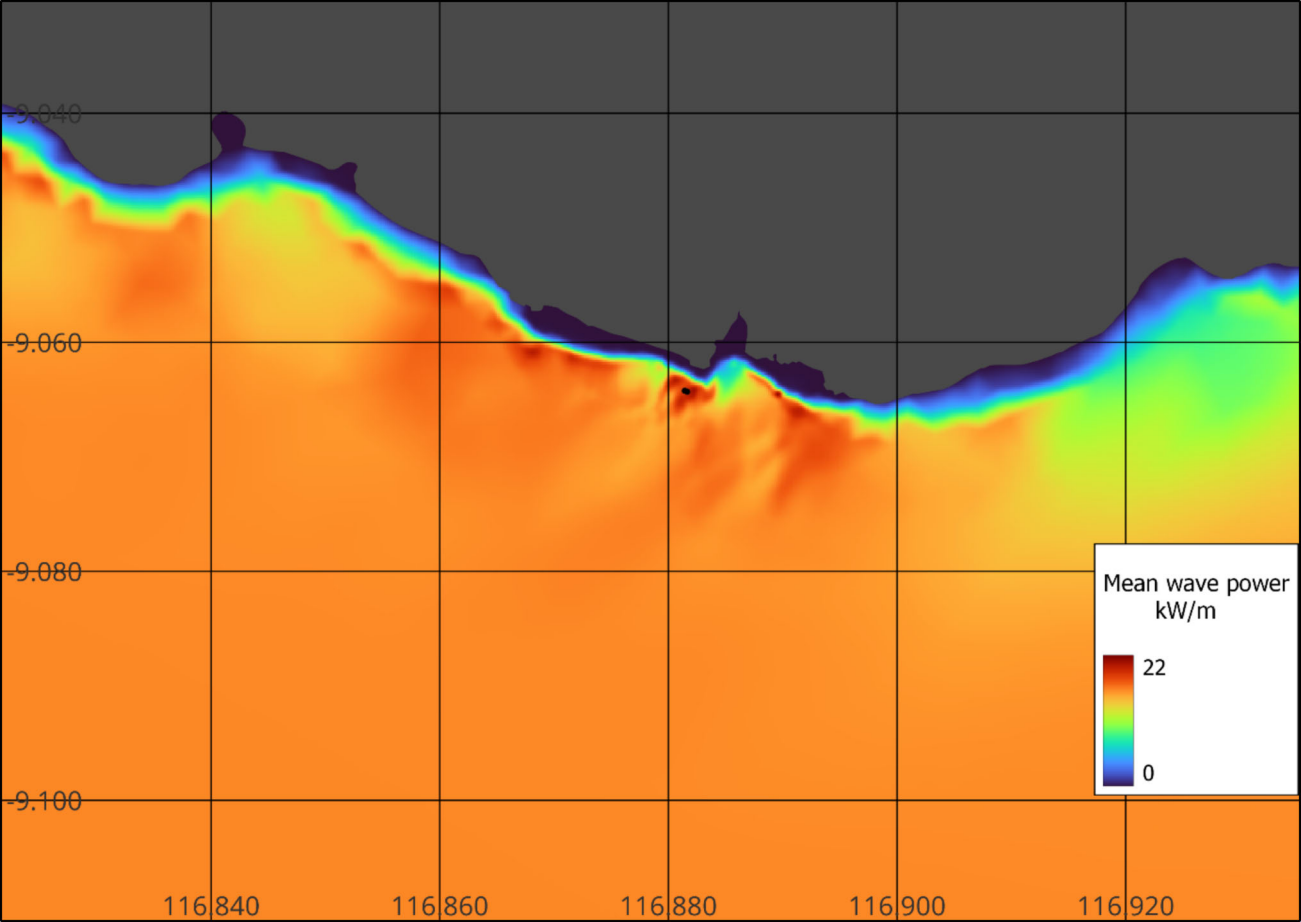
High-resolution TOMAWAC model results, 10-year mean wave power. Proposed position of WEC buoys shown as small black circles. Additional wave energy flux maps are presented in the Supplementary Information

High-resolution model results included wave height, peak period, breaking coefficient, direction, frequency, and mean period at each mesh node (Fig. 9). In contrast to the WAVERYS results, the TOMAWAC wave model shows variation of wave energy along the coastline due to the redistribution of wave energy. The 10-year mean indicates the site of interest experiences elevated wave energy due to the focussing and redirection of wave fronts caused by the local bathymetry and the curvature of the coastline, which together amplify wave intensity through constructive interference and energy convergence. In addition, the wave energy seasonally varies from a minimum of 12 kW/m in January–February to a maximum of 26 kW/m June through August for a 10-year monthly mean (Supplementary Information).

**Fig. 10.**
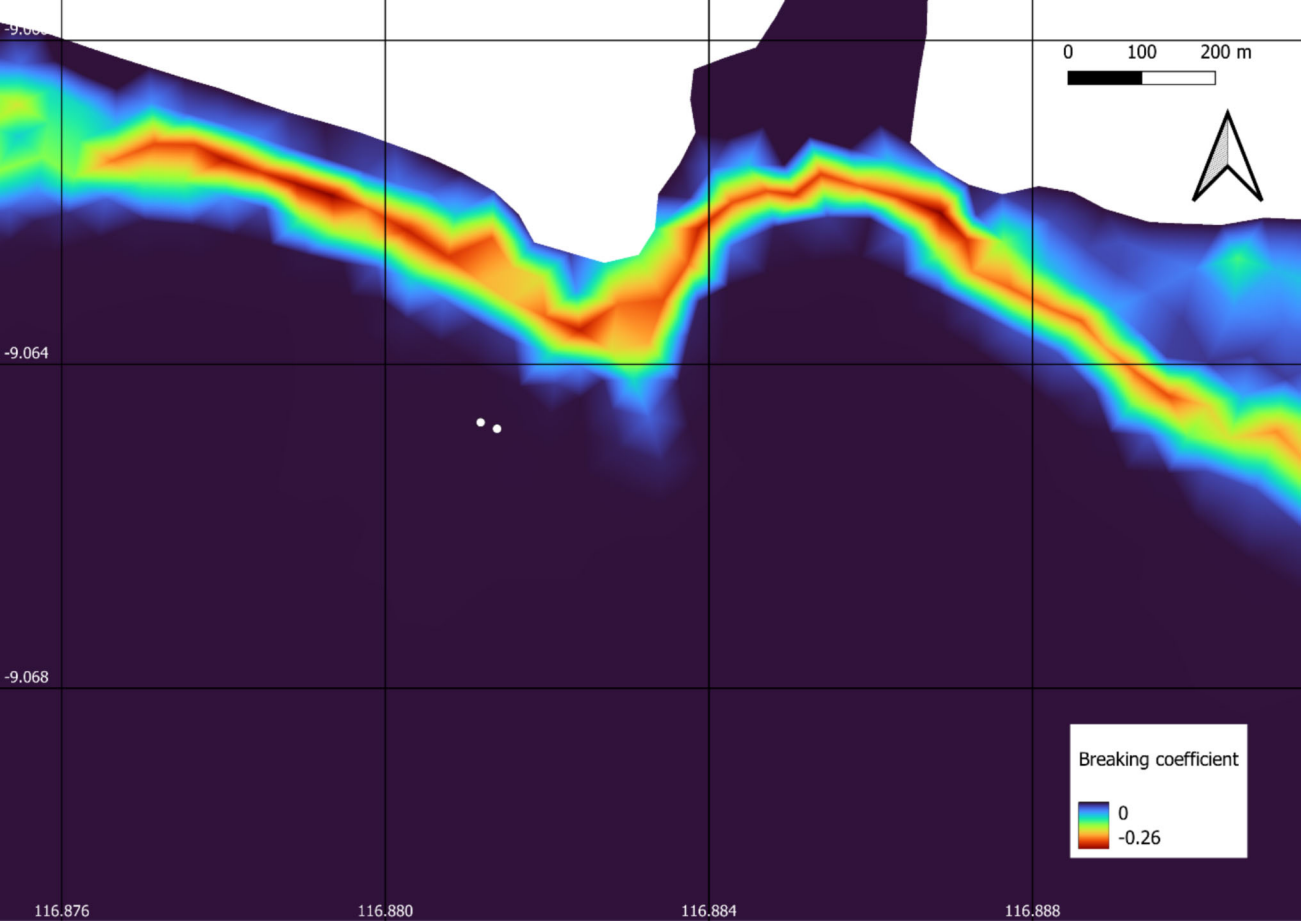
10-year mean wave breaking coefficients, where a negative value indicates loss of energy due to wave breaking (unitless). The two white circles indicate proposed WEC buoy positions

The TOMAWAC model results were valuable in assessing whether the proposed WEC locations fell within the typical wave breaking zone—an undesirable placement due to high turbulence and structural risk—whilst still being sufficiently close to shore to allow for convenient maintenance access. The wave breaking coefficient ranges from 0 to − 1 and indicates how aggressively wave energy is dissipated, with values closer to − 1 indicating loss of energy due to wave breaking. Whilst wave breaking has not been calibrated for this model against observational data from the site, based on default wave breaking coefficients (breaking coefficient = 0), the results indicate that the proposed position should be outside the normal breaking zone (Fig. 10).

## Discussion

Comparison between the altimeter-derived significant wave height and the WAVERYS global model-derived significant wave height showed low bias in the extreme offshore of the area of interest (0.08 m Hs, approximately 200 km from shoreline, Figs. 4, 5) to a bias of 0.23 m Hs closer to the shoreline and edge of the high-resolution model (18 km from the shoreline, Fig. 6).

For the high-resolution model area, there were 77 AltiKa data values. For one value (14/7/19), close to the shoreline (-9.1640°,116.806°), there was a large difference in significant wave height values (1.09 m), where the model provided a value of 2.07 m Hs, and the corresponding altimeter value was 3.16 m Hs. For the remaining values, the maximum bias was 0.3 m. As the point was 14 km from the shoreline, the reason for the large difference in one point is unknown. It is possible a short period of increased wave height could have been missed by the boundary parameters supplied by the global WAVERYS model which was detected by the altimeter. The time measurement between the satellite measurement and the model is < 15 min and bathymetry deep enough as not to affect the waves. One potential influence may be some bias of the satellite measurement at the time of measurement. In the absence of additional comparisons or calibrations, the comparison to altimeter data, whilst limited, still provides some confidence in using the model data as an estimate for high-resolution area potential wave energy calculations over a 10-year period, including seasonal variations. These results have high correlation with the satellite data and are comparable to wave model validation statistics found elsewhere (Alday et al. 2022; Zhang et al. 2023).

The plots from the global WAVERYS data suggested that the wave power is seasonal with the highest wave power available during July and the minimum wave power available during December and January. Results generally agree with the previous literature on Indonesia wave power potential, for example Ribal et al. (2020). The seasonal wave characteristics along the south coast of Indonesia are strongly influenced by the tropical monsoon climate (Kuntoro et al. 2020).

Results of the high-resolution model show wave energy redistribution along the coastline. This leads to a higher wave energy at the site of interest than some other areas close by (Fig. 9). The redistribution of available energy results from the coastline’s shape and bathymetry, concentrating wave energy in specific areas, including the site of interest—a headland—and the result of dominant wave directions. The model indicates that the wave direction and periods are favourable for a WEC device, with a narrow window of wave directions, with the most frequent waves 1.5–2 m in significant wave height and 10–11 s in peak period over a 10-year period. The model suggests that the proposed locations for WEC buoys are optimally positioned in terms of both wave energy (Fig. 9 and Supplementary Information) and wave breaking (Fig. 10) showing seasonal lows of greater than 10 kW/m, optimal for the WEC considered in this study. Although in-situ buoy data are recommended to calibrate such models, for this initial study with limited resources and data in a remote location, this high-resolution model with low-cost derived bathymetry compared well with satellite-derived significant wave heights.

**Fig. 11:**
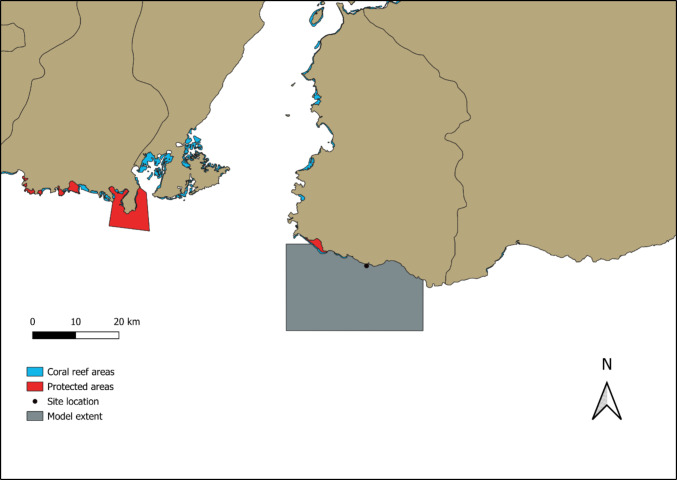
Coral reef mapped from satellite data around the area of interest. Data; UNEP-WCMC, WorldFish Centre, WRI, TNC (2021) and UNEP-WCMC, and IUCN (2024)

Besides the coastal shape, coral reefs are another common factor along this coastline (as shown in Fig. 11), acting as a barrier to wave energy (Ferrario et al. 2014). Additionally, although the site of interest for this project is not affected, certain coastal areas are designated as protected zones. In addition to GIS-based studies identifying promising locations for potential small-scale wave energy converters, high-resolution modelling following the methods here could provide a low-cost estimation of wave energy at specific sites of interest. Such sites could be identified through available GIS layers of energy infrastructure, protected areas, and leisure areas, amongst other considerations (e.g., Purba et al. 2015).

## Conclusion

The model results revealed significant potential for wave energy conversion at the site of interest, both seasonally and over a 10-year period. The high-resolution model also highlighted substantial variation along the coastline due to local bathymetry and coastline shape, resulting in energy redistribution. Based on these findings, initial design parameters for the wave energy converters (WECs) could be derived, along with valuable information to guide micro-siting the WEC buoys.

Comparisons between the high-resolution model and altimeter data indicated that the model exhibited an acceptable bias for the study’s purposes. This type of low-cost modelling could provide valuable information for other promising sites of interest along the coastline of Indonesia with the potential of attracting investment from developers or guiding more detailed site characterisation.

The study conducted on the southwest shoreline of Sumbawa Island in Indonesia’s West Nusa Tenggara province has demonstrated significant potential for the deployment of small-scale wave energy converters, based on current infrastructure, energy requirements, and available wave energy shown in this study. The research highlighted the abundant wave energy resources available along Indonesia’s extensive coastlines, which could be instrumental in addressing energy security, environmental, and economic challenges. By leveraging the high-resolution wave model developed for the site, the study also provides a valuable case study and methodology for assessing renewable energy resources in remote areas.

The use of both global and high-resolution models has allowed for a comprehensive analysis of wave energy availability, despite the lack of in-situ wave data. The innovative approach of combining low-cost bathymetric surveys with available data and satellite altimetry has proven effective in overcoming these limitations, although site-specific dependencies, such as satellite coverage, accessibility for local surveys, and availability of high-resolution bathymetric data, should be considered when considering applicability to other sites. The selected site near Hasama Cliff, Punak beach, offers favourable conditions for the WEC installation, with considerations for infrastructure, energy demand, and accessibility investigated in wider studies.

This research underscores the importance of renewable energy development for Indonesia, particularly in remote regions like Nusa Tenggara, where electrification and infrastructure investment pose significant challenges. The successful implementation of WECs could provide a sustainable solution to these issues, reducing reliance on diesel generators, improving air quality, and contributing to the global effort to decarbonise energy systems.

The study’s findings are promising for the future of marine energy markets in Indonesia and similar archipelagic nations. The potential for creating new jobs and significantly reducing CO_2_ emissions through the adoption of wave energy is a testament to the transformative power of harnessing nature’s forces for a sustainable future.

## Supplementary Information

Below is the link to the electronic supplementary material.

Supplementary file1 (PDF 461 KB)

## Data Availability

Datasets generated during the current study are available from the corresponding author on reasonable request.
