# Supplementary material for: Methodology for wave power estimation at remote sites with satellite altimeter validation applied to Indonesia
Source: J Ocean Eng Mar Energy. 2025 Dec 8;12(2):661–74. doi: 10.1007/s40722-025-00455-0 (PMC13095939; doi:10.1007/s40722-025-00455-0)
Supplement: Supplementary file 1 — Supplementary file1 (PDF 461 KB) [file 40722_2025_455_MOESM1_ESM.pdf]

Methodology for wave power estimation at remote sites with satellite altimeter validation applied to Indonesia – Supplementary Information

Jason McIlvenny[1], Benjamin J Williamson[1*]

[1] University of the Highlands and Islands, Inverness, UK
*Corresponding author: benjamin.williamson@uhi.ac.uk
Jason McIlvenny 0000-0002-5342-7003
Benjamin J Williamson 0000-0002-7107-7713

WAVERYS Wave Power maps (10-year means)

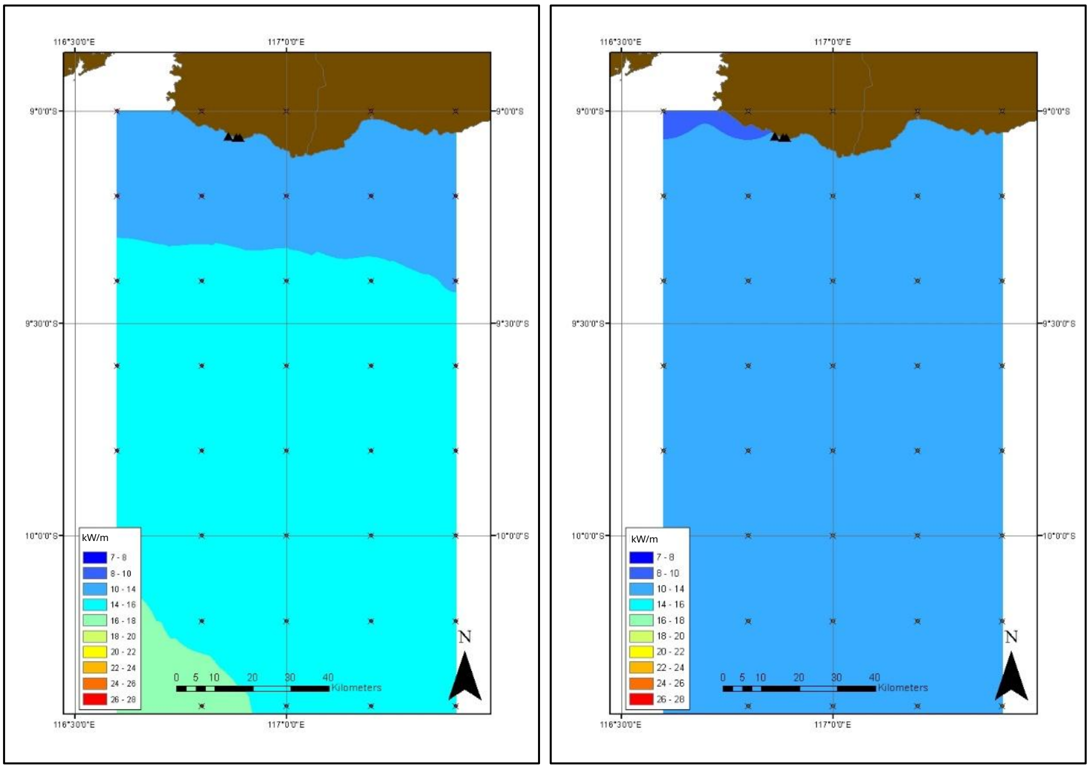

WAVERYS Wave Power maps (10-year means) January, March

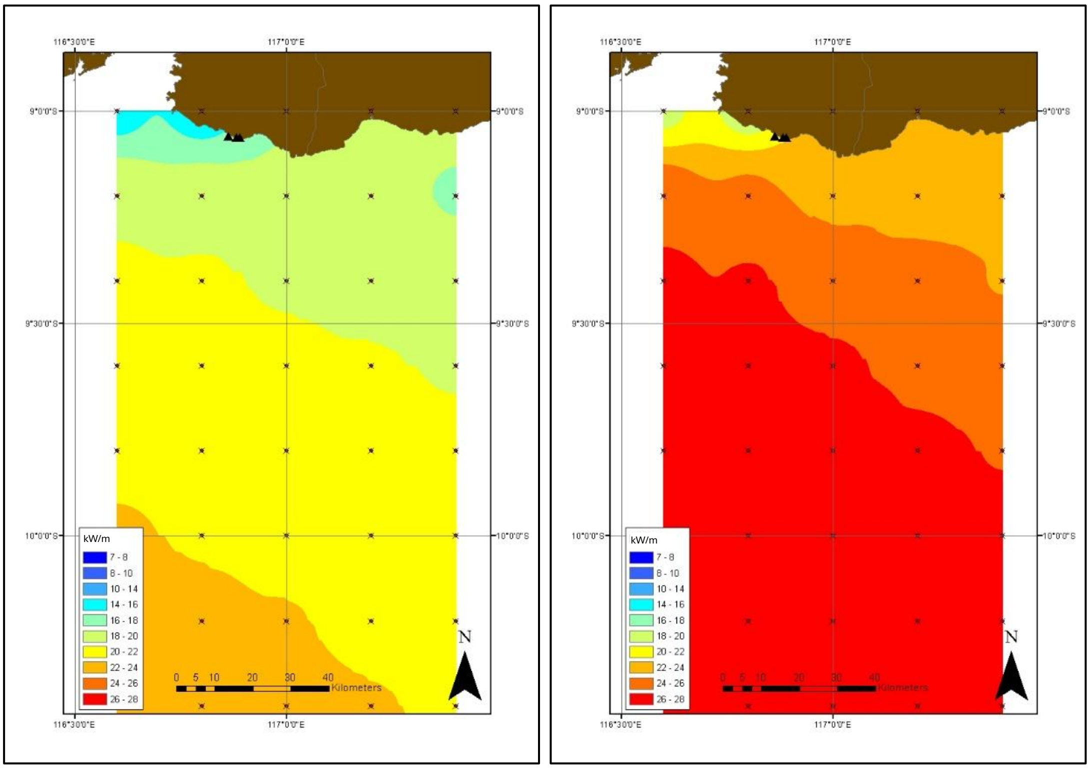

WAVERYS Wave Power maps (10-year means) May, July

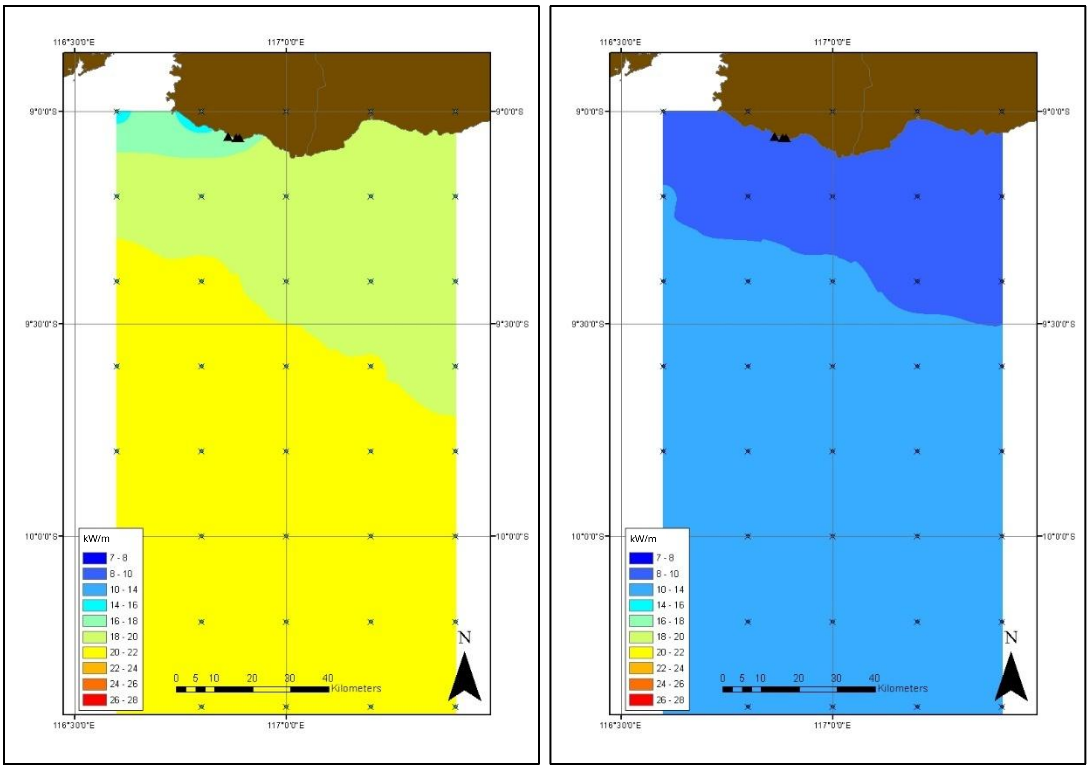

WAVERYS Wave Power maps (10-year means) September, November

TELEMAC-TOMAWAC Wave Power results (10-year means)

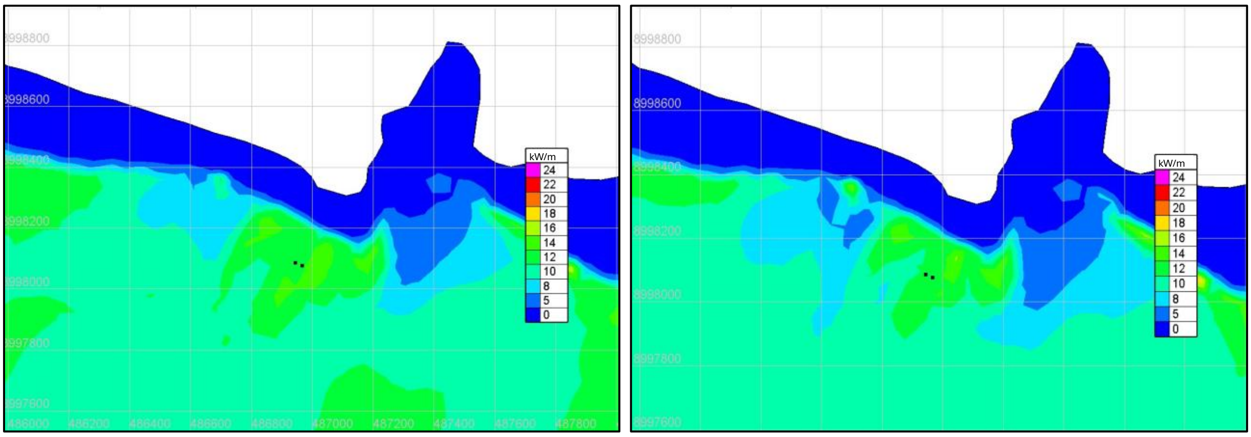

TELEMAC-TOMAWAC Wave Power results (10-year means) January, March

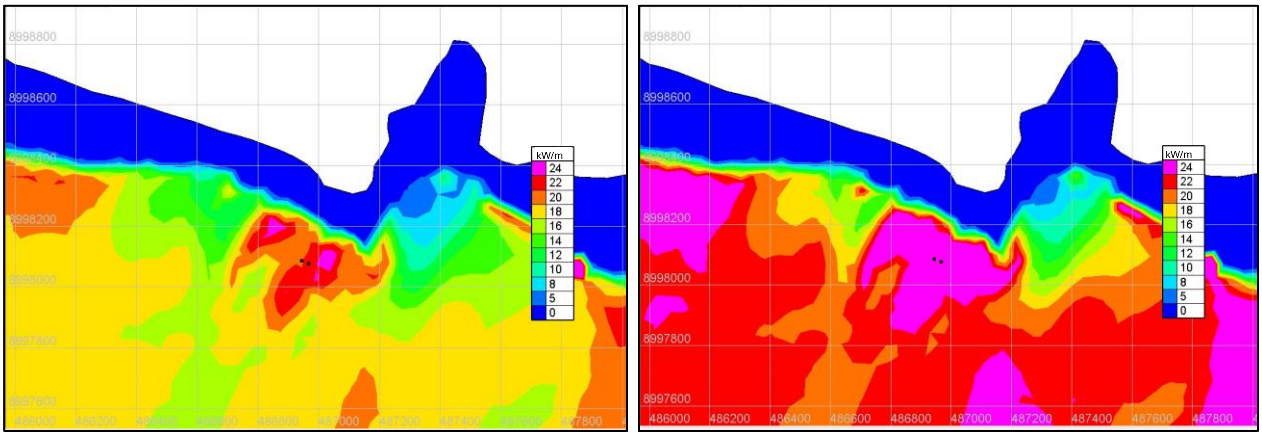

TELEMAC-TOMAWAC Wave Power results (10-year means) May, July

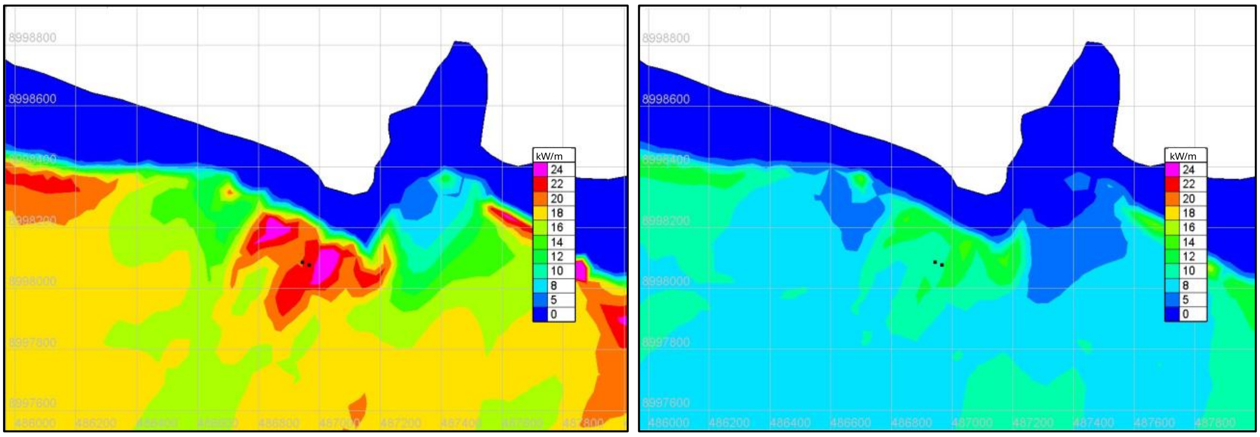

TELEMAC-TOMAWAC Wave Power results (10-year means) September, November
